# Asset-Based, Sustainable Local Economic Development: Using Community Participation to Improve Quality of Life Across Rural, Small-Town, and Urban Communities

**DOI:** 10.1007/s11482-022-10051-1

**Published:** 2022-06-22

**Authors:** Matt Kammer-Kerwick, Kara Takasaki, J. Bruce Kellison, Jeff Sternberg

**Affiliations:** Bureau of Business Research IC2 Institute, The University of Texas, Austin, USA

**Keywords:** Sustainable Community Development, Community Participatory Research, Assets, Values, Identity, Quality of life

## Abstract

We analyze survey data from 5,487 residents of 85 Texas communities, including rural communities, small towns, medium-sized cities, and the five largest urban centers, to model the influence of the degree of urbanity and rurality of a community as well as residents’ social position, values, and their satisfaction with local assets. The paper’s central argument is that a wide range of factors impact how community members think about their needs and thus should be considered in any community development process. This human-centered approach looks at how community specific factors impact economic and quality-of-life development project preferences in support of adapting community development decision-making processes according to the context of each community. For the sake of parsimony, this paper presents results for three community development project concepts: (1) renovating some downtown buildings as mixed-use facilities with retail shops and apartments, (2) opening a community health center, and (3) deploying gigabit high-speed fiber broadband internet in the downtown area. Our findings specifically highlight areas where participatory processes can be introduced to guide further interactions between experts and community members to (1) identify groups in the community to bring into the process who may be most impacted by the choice of one intervention over another, and (2) target areas where further community discussion and deliberation is necessary around which community dialogues (round tables, town halls, workshops, etc.) could be facilitated.

**Supplementary Information:**

The online version contains supplementary material available at 10.1007/s11482-022-10051-1.

## Introduction

People who want to improve quality of life in the place where they live must often make choices constrained by other residents’ preferences and priorities about which projects have the best fit for their shared community. With fewer resources, smaller, more rural communities often have less room for error. Moreover, the migration of more educated workers and prime working-age residents from rural to urban areas puts economic stress on rural communities (Domina 2006; Kilkenny 2010; Parker et al. 2018).

Researchers have studied social, cultural, and political divisions between urban and rural residents who are thought to have fundamentally different types of personalities, values, cultures, politics, and needs (Brown & Kandel 2006; Gimpel et al. 2020; Kellogg Foundation 2002). These differences in the social, cultural, and political characteristics of urban and rural America have been chalked up to the relative isolation of rural communities from the social and cultural influences of the city, seen as a preserve of traditional American values (Logan 1996), or a stagnant cultural backwater of reactionary conservativism and underdevelopment (Lewis 1998). Each type of settlement is thought to either produce or reinforce different personality types, whose interaction in these spaces produces sets of values and cultural frameworks that evaluate the world differently, which in turn make different types of decision-making, actions, and outcomes possible over others.

Yet, studying quality of life through an urban-rural binary is a simplification of the way that regional contexts develop. Schema for classifying a community as urban or rural abound and change over time (for a review see National Academies of Sciences and Medicine, 2016, Appendix B). Communities can be more accurately seen as possessing a degree of urbanity and rurality by incorporating multiple dimensions of information that include local population size, local population density, and commuting distance to sources of employment, healthcare, culture, and other forms of entertainment.

We examined these issues formally by addressing the following research questions:

1. How, if at all, do community size, population density, and distance to a major metropolitan area predict preferences for and priorities among various community development project concepts?
2. How do community members’ social position (for instance, age, race, income) predict preferences for community development project concepts?
3. To what degree do personality types and personal values help predict preferences for community development project concepts?
4. To what degree does community satisfaction with and the perceived importance of assets available in a community further improve predictions about preferences for various community development projects?

To answer these research questions, we present the findings of a survey fielded in the summer and fall of 2020 (during COVID-19) among Texas residents that asked participants to allocate hypothetical “points” toward 6 distinct economic development project concepts for their town (for a rural resident) or neighborhood (for a big-city resident). We analyze survey data from 5,487 residents of 85 Texas communities, including rural communities, small towns, medium-sized cities, and the five largest urban centers to predict respondent economic and quality-of-life priorities based on a community’s degree of urbanity and rurality and individual respondents’ social position, community values, personality types, and community asset satisfaction and importance. By using a participatory process to consider what members of different communities would choose for their communities to invest in using a trade-off exercise, we put the human and community member view of their needs first in determining which projects should be prioritized in any given community, looking at how each community member sees the connected needs of the community and how they impact each other. This human-centered approach to looking at how these factors impact economic and quality-of-life development project preferences will support the adaptation of community development decision-making processes to the conditions of each community.

## The Role of Community and Quality of Life in Economic Development

Economic development theories and practice commonly address sustainability across multiple objectives and decision-making criteria. Sustainable Local Economic Development (SLED) is a model that incorporates the community into the development process with a focus on producing equitable long-term solutions (Newby 1999). SLED’s concern with sustainability comes from its critique of traditional economic development practices, which viewed development, and the assets and values that it is based on, from the narrow view of economic actors (external capital and local elites) operating outside of any given community. Traditional development approaches were performed by elites and technicians who viewed a community as a source of labor, infrastructure, and raw materials for supporting a given type of economic or business activity. In the traditional model, communities that wanted economic development presented themselves through the previous lens to attract outside capital because any economic activity or growth placed in a community was believed to be a net good (Vaughan, Pollard & Dyer 1984).

SLED starts from the basis that economic activity and growth may not be good for its own sake; economic development must also concern itself with how economic activity impacts the lives of the communities incorporated into it. SLED holds that the needs of community members should be considered in the economic development decision-making process. The community needs should be used to guide selection over which type of development intervention should be chosen based on equitably balancing trade-offs between outside capital and the community, asking whether an intervention or project improves the future quality of life, equity, participation, and partnership in the community, even if privileged or institutional economic actors were to end their involvement.

Asset-Based Community Development (ABCD) moved beyond SLED to frame a community not just as a partner to economic development but as a system of subsystems, the economy being one of many subsystems, that needs to be developed to maintain a balance or equilibrium (Nel 2018). ABCD addresses these subsystems broadly, including rights, capacities, and capabilities across human, physical, financial, natural, political, social, spiritual, and cultural assets (or capitals). ABCD is guided by the principles that everyone has gifts, relationships build a community, leaders involve others as active members of the community, people care about something, and that people can be motivated to act. These principles and the ABCD approach operate to mobilize 5 key assets: individuals, associations, institutions, physical assets, and connections (Collaborative for Neighborhood Transformation 2020). From this basis, Asset-Based Community Development starts at the level of the individual, who is thought to have their own talents and assets to mobilize. The mobilization of these assets toward meeting shared community needs relies first on the individual identifying and evaluating their own assets and needs, and from there those of their community. The individual then finds motivation to mobilize these assets and forms connections with other motivated community members to create associations and wield institutions to connect different communities to meet shared needs.

We focus on a human-centered, participatory approach that builds from SLED and ABCD by attempting to start from the needs of the community as a way of assessing projects that would meet the human needs of the members of that community. Good jobs, human flourishing, and community creation are as important—if not more important—than economic growth. This framing of a human-centered approach to sustainable community development draws from the literature on human-centered design, wherein product solutions are created with the involvement of the human perspective at all steps in the planning and development process. However, this approach is less commonly applied to psychosocial interventions and implementation strategies (Lyon, Brewer, & Areán 2020). Here, the quality of life of the community is a way to plan for sustainable and locally appropriate economic development that requires individual-, community-, and economy-level information. Quality of life serves as a domain of observation from which the balance between economic and community concerns can be measured. Individual needs have been shown to be shaped and impacted by individual personality type and personal values, their individual well-being, and levels of perceived community attachment and community satisfaction (Fried 1982, Theodori 2009). Community-level needs have been shown to be shaped by individual-level needs, and all the factors that are associated with them, as well as by the underlying assets of a given community, levels of community satisfaction with these assets, and the shared culture and values held by individuals coming together as a community (Fried 1984, Hanscott 2016, Garretsen et al. 2019, Stinner and Van Loon 1992). As McGregor, Camfield and Woodcock (2009) find, people identify their needs and interpret their quality of life through a local context, which suggests that economic development should not only assess satisfaction with a community asset but also the importance of that community asset to its community members.

## Rural and Urban Community Personality, Values, Satisfaction, and Decision-Making

Settlement type was the starting point for American Urban Sociology (Park, Burgess, McKenzie 1926) which built from competition and succession models from studies of natural ecology to understand how human behavior is shaped by social structures and factors of the physical environment. This school of thought argued that the ecological pattern of a community – the spatial distribution of its economic, cultural, and social relationships within and among groups – has independent effects on social life separate from those of other explanatory variables. Georg Simmel (1950) was among the first to theorize the impact of settlement type on personality. Simmel argued that rural and urban settings established different conditions for the creation and flourishing of distinct personality types. Rural settings were thought to provide relatively stable, predictable, and habitual ways of life and interaction, with residents not often experiencing change or new kinds of stimuli. When new stimuli were encountered, it would be jarring and reacted to rashly, causing this rural personality type to want to avoid change. This type of personality can be seen to correspond with values of conservativism and tradition. In urban settings, new stimuli were encountered constantly, due to the city’s population size, density, and heterogeneity (Wirth 1938), producing high levels of mental energy and stimulation. This type of personality characteristic of urban settings is thought to adopt a jaded and dispassionate stance toward their condition of ever-present instability, tolerating and embracing change as a constant in life, leading to values of openness and progressivism.

Through personality type, urbanity and rurality have been shown to have an impact on community satisfaction, as a product of whether the personality of an individual matches those of the people around them, and whether the location they are in, both in terms of the compilation of personalities clustered within it, and the contours of the community itself, allows that personality to thrive (Fried 1984). It has been shown that cities have different compositions of personality types, which lend themselves to more or less of an entrepreneurial culture, which in turn impacts their economic performance and growth (Garretsen et al. 2019). The physical, social, and cultural assets of a community, and its level of social inequality relative to other communities, have also been shown to have a direct and primary effect on levels of residential and community satisfaction (Fried 1982). The types of assets available in a community and their quality are thought to vary across the urban-rural divide, with small rural communities believed to have more responsive local governments, greater social solidarity, and more pleasing physical environments. In contrast, larger cities are thought to have greater economic opportunities, cultural environments, and more highly developed public service infrastructure (Wilkening 1982; Stinner & Van Loon 1992).

Community satisfaction has been shown to have an impact on decision-making in both urban and rural circumstances and has also been used to evaluate the success or failure of different planning initiatives. One study showed that community satisfaction with various community assets and community size preferences are strong predictors of decision-making when it comes to migration intention, this decision-making also influenced by metropolitan versus non-metropolitan residence and whether the migration intention was short-term or long-term (Stinner & Van Loon 1992). The influence of planning interventions on growth rates and community satisfaction across the urban-rural continuum has also been studied, with Baldassare et al. (1982) finding that places that adopted growth controls had lower community satisfaction; however, the impact of community satisfaction on the selection of planning interventions and their outcomes has yet to be studied.

Degree of urbanity and rurality can cause variation in factors (personality type and values, community satisfaction) connected to decision-making processes in general, and community development in particular; however, the relevance and primacy of the interaction effect of settlement type on these factors, and decision-making as a whole, remains an open question. Lichter & Brown (2011) argue that perceptions about urban and rural communities do not reflect the reality on the ground, with many scholars (Hamilton 2006, Weber et al. 2005) claiming that differences along the urban-rural continuum may have less to do with settlement type than with spatial differences between urban and rural residents such as social boundaries within spatial groups (race, class, gender, etc.). The following analysis will test whether settlement type has an independent effect on decision-making when it comes to community development preferences.

## Method

We compare Texas resident responses in mixed multi-level logistic regression models to explain outcomes of a community investment allocation exercise across economic development and quality-of-life development concepts. We include community size, community population density, and distance of the community from the nearest major urban center to assess the influence of the degree of urbanity and rurality. We include basic human values as well as the personality traits of agreeableness and openness to assess the influence of underlying psychological traits of members of communities. And, we include community members’ satisfaction with and the perceived importance of community resources to assess the influence how community members think about resources that are available to them. As controls, we also analyze the effects of demographic variables and perception of emergent COVID-19 impacts on the resident’s community. We hypothesize that respondents’ values and level of agreeableness would predict—given the constraint of finite resources—preferences for economic and quality of life interventions. We thought choosing and allocating resources to interventions would be further explained by the degree of satisfaction with what is currently available as well as how important those resources are to the community.

### Measures

#### Schwartz Value Theory

Schwartz’ theory of basic human values (Schwartz 2012) argues that all cultures are structured by a set of ten distinct personal values. People use values to motivate action toward goals. These goals help people to deal with the universal requirements of being humans who live with other humans. The ten values consist of self-direction, stimulation, hedonism, achievement, power, security, conformity, tradition, benevolence, and universalism. These values form a circular motivational continuum that reflects the conflict and compatibility among values.

Schwartz theory argues that the continuum is organized along two bipolar dimensions, where “openness to change” and “conservation” are compared in one dimension, and “self-enhancement” and “self-transcendence” are compared in another. For example, pursuing the value of self-direction—autonomous control over one’s life—in the dimension of openness to change, would conflict with pursuing the value of conformity—group harmony—in the dimension of conservation. Another example would be pursuing a value that upholds the interests of others in the dimension of self-transcendence, like universalism, conflicting with pursuing the value of power, which values dominance over others, in the self enhancement dimension.

To model the impact of personal values across these bipolar dimensions on community decision-making preferences, we have incorporated resultant self-transcendence (self-transcendence – self enhancement) and resultant conservation (conservation – openness to change) into our survey. This approach is common and has been used in a variety of marketing and business strategy settings (Ahmad, et al. 2020; Ashraf, et al. 2020; Keh and Sun 2008; Steenkamp, ter Hofstede and Wedel 1999).

#### Big Five Personality Traits

The Big Five Inventory (BFI) measures five personality dimensions that are theorized to be and have been tested to be relatively stable and distinct over the life course and applicable across cultures (Cobb-Clark & Schurer 2011; Schmitt et al. 2007; Soldz and Vaillant 1999). These personality dimensions are measured in a way that precludes the need to break down these personality dimensions into lower order measures (John et al. 1991). We measured agreeableness and openness because the research suggested that these personality dimensions would predict a respondent’s likelihood to invest in a community intervention. For reasons of parsimony and to avoid survey participant fatigue, we did not include conscientiousness, extraversion, and neuroticism from the BFI.

*Agreeableness.* From the Big Five Inventory (BFI) we hypothesize that variation in agreeableness would be the personality factor that would best predict whether community members *would cooperate toward an intervention.* Agreeableness is correlated with wanting to engage in positive actions for society. Agreeableness is positively related to the orientation of Self-Transcendence and to the individual value of Benevolence from Schwartz’ value theory.

*Openness*. Openness to experience is a personality trait whose distinct facets can be broken down into three higher-order measures of intellectual curiosity, active experiencing of senses and emotions, and open mindedness toward different cultural ideas and values (Christensen et al., 2019). Central aspects of openness distinct from other personality traits include willingness to entertain novel ideas and unconventional values, intellectual curiosity and reflection about the inner and outer world, and independent judgement. Openness to experience has been studied in relation to creativity and innovation in social entrepreneurship (Nga and Shamuganathan, 2010), adjustment to change, identification and maintenance of specific communities (Chang, et al. 2013; Füller, et al. 2008), and engagement with community development interventions (Litchfield & Javernick-Will 2015). Based on this research, openness to experience seemed to be a personality trait that might predict respondent likelihood to invest in community development interventions.

#### Asset Satisfaction and Importance

We asked survey respondents to rate how satisfied they were with eighteen assets of their community, on a scale from 1 = not at all satisfied to 7 = extremely satisfied. Participants were also asked to assess the importance of these same eighteen assets if they were to be considering relocating to another community (1 = not at all important to 7 = extremely important). The eighteen community assets consist of the following items: broadband internet, cellular or mobile telephone options, arts and culture options, nature and outdoor options, walking and biking options, public transportation, infrastructure conditions, institutions of higher education, primary and secondary education of children prior to college (referred to as K-12 education), housing affordability, housing availability, employment options, cost of living, incentives to start or expand a business, library, healthcare, childcare options, and safe environment.

We expected and confirmed multicollinearity among the community assets. We used exploratory factor analysis (EFA) and reliability analysis to create four mean asset satisfaction subscales from the original eighteen survey items, previously listed, see Table 1. Principal components analysis with varimax rotation (KMO = 0.916, sig. < 0.001) produced asset themes for broad-based community services, economic environment, family-oriented community services, and communications services with 40.8%, 9.2%, 6.8%, and 6.0% variance explained for each, respectively. Average asset scores were defined for this grouping of four asset themes, with reliability analysis adequate for all subscales, Chronbach α > 0.75. Reliability was not improved by dropping any asset from the subscale characterized by the EFA. We used these same four themes to calculate asset importance subscales.

**Table 1:** Community Asset Satisfaction Subscales

Broad-based community services (Chronbach’s α = 0.85)
• Arts and culture options
• Nature and outdoor options
• Walking and biking options
• Public transportation
• Infrastructure conditions
• Institutions of higher education
Economic environment (Chronbach’s α = 0.85)
• Housing affordability
• Housing availability
• Employment options
• Cost of living
• Incentives to start or expand a business
Family-oriented community services (Chronbach’s α = 0.80)
• K-12 education
• Library
• Healthcare
• Childcare options
• Safe environment
Communications services (Chronbach’s α = 0.78)
• Broadband (internet)
• Cellular or mobile telephone options

#### Additional Explanatory Variables

We include other variables that may explain partial variance in intervention interest. These variables include community size, population density of each community, and the distance of each community to the nearest major metropolitan center in Texas. We also include community as a random effect in our analysis because of our multi-site data collection design. We include the age, race, gender, education, and income of community members in sample to assess the role of social position. Lastly, this study was conducted during the COVID-19 pandemic, so we include variables indicating participants’ perception of how COVID-19 impacted their community’s health and economy.

#### Interventions

In a point-allocation exercise, we examined how community members prioritize and make trade-offs among a set of community development project concepts. The concepts chosen for this manipulation are all popular options both considered by and implemented in various community settings (see, for example, Hightree, et al. 2018). We employed very simple descriptions of these community development concepts in this survey, but the concepts were selected based on need expressed in literature as well as from comments obtained during qualitative exercises conducted during the planning phase for this survey.

The allocation exercise asked the participant to “*allocate 100 points across the following projects based on how well you think they would fit the needs of (insert resident’s community). Allocate more points to projects that you think are a better fit for (insert resident’s community). If a project does not have any fit, allocate 0 points to it. If only one project fits (insert resident’s community)’s needs, allocate all 100 points to that project.*” The seven intervention options included the following: Renovating downtown buildings with retail shops and apartments, opening a community health center, deploying high speed internet downtown, adding more computers and meeting spaces in the public library, early college credit and vocational programs for high school students, opening a co-working and startup working space for entrepreneurs, and an “other” category to capture additional significant intervention options. The wording included the phrase “in my neighborhood” for surveys conducted in the 5 major metro areas to localize the context. For example, the first option was worded “renovating some buildings in my neighborhood as mixed-use facilities with retail shops and apartments.”

For the sake of parsimony, the present study presents results for three community development project concepts. The results for all seven concepts are available from the corresponding author. We selected renovating some downtown buildings as mixed-use facilities, opening a community health center, and deploying gigabit high-speed fiber broadband internet downtown as the concepts to focus on in this paper because of their relevance in general in our literature review and to the communities included in our study, as will be discussed further in our findings. The motivational context for the chosen concepts is discussed next.

*Renovating some downtown buildings as mixed-use facilities*. We have observed downtown renovation efforts in small towns in New Hampshire; Lufkin, TX; Sweetwater, TX and Del Rio TX, among others. The benefits of a town center even extend to urban neighborhoods. Specifically, Pendola & Gen (2008) demonstrated that urban neighborhoods with a “main street” are endowed with a higher sense of community than other urban and suburban neighborhood settings. Gibson, Zurcher, & Wisemiller (2020) show that direct public-private investment in downtowns of smaller towns has a spillover effect on additional private investment in renovations and maintenance in the downtown area. Powe (2020) compares strategies that have been used successfully by communities, recommending that town centers be thought of as “complex adaptive places, their multi-functionality must be treasured and recognition given to the unpredictability/serendipity of opportunities emerging within them.” By renovating downtown to include mixed-use buildings there is an opportunity to increase walkability of the area and increase social capital between residents, thereby also increasing their quality of life (Rogers, et al. 2011). This intervention is also important to assess in our gradient of urbanity and rurality because Jeffres et al. (2009) found that when people believe they can access “third places”, defined as places where people can meet and talk outside of home and work, they perceive a higher quality of life in their community.

*Opening a community health center*. Community health centers (CHCs) were launched in 1965 during President Lyndon B. Johnson’s War on Poverty. CHCs were designed to reduce health disparities experienced by racial and ethnic minorities, the uninsured, and the poor. They provide primary medical, dental, behavioral, and social services to medically underserved populations in medically underserved areas, including migrant and homeless populations that do not have the ability to pay. CHCs have received substantial bipartisan legislative funding to address a growing need for primary care (Adashi, et al. 2010). Their presence in a county has been shown to lower the rate of hospitalization for ambulatory care sensitive conditions among older adults and in some cases, for working-age adults as well (Probst, et al. 2009). CHCs are models of community oriented primary care and especially serve populations facing language and culture barriers that may need additional translation, interpretation, and transportation services. CHCs are an opportunity for community development by leveraging local resources among critical access hospitals, rural health clinics, and educational institutions (Geiger, 2002; Samuels et al., 2008).

*Deploying gigabit high-speed fiber broadband internet downtown*. The disparities in access to high-speed internet between urban and rural communities are widely discussed in the media (e.g., CNN’s “America’s surprising breeding ground for inequality: The internet,” May 17, 2020) and by researchers (e.g., Pew Research Center’s Internet/Broadband Fact Sheet). These disparities are also central to the Biden Administration’s The American Jobs Plan (The White House 2021) wherein high-speed broadband is considered a fundamental infrastructure along with roads, drinking water, and electricity. Indeed, we have observed Smithville, a small community in Texas, deploy fiber along its main street as a central part of an economic development program intended to attract new business to its community. Other communities have done the same, including Tullahoma, TN; Mount Washington, MA; and Wilson, NC, just to name a few (Hanna & Mitchell 2020).

## Procedure and Participants

Data for this paper were collected in two phases, both of which occurred in 2020 during the COVID-19 pandemic. A purposeful community sampling methodology was used to collect data from 80 rural and small-town communities outside of the 5 largest metro areas in Texas (consisting of Dallas/Fort Worth, Houston, Austin, San Antonio, and El Paso). Student researchers promoted the project and distributed links to the web-based survey in rural and small-town communities using a variety of techniques, including social media; telephone calls to local community members and leaders; and distributing electronic fliers to local groups to post on their websites. These communities were incentivized with funding for school computer equipment to be donated to the community with the highest number of completed surveys per capita. Participants from the 5 largest metro areas in Texas were recruited through a commercial panel, Dynata.^1^ The rural and small-town surveys were collected during the summer of 2020 while the major metro surveys were collected during the fall of 2020. The study was reviewed and approved by authors’ institutional review board.

We analyze survey data from 3,363 residents of the rural and small-town communities and 2,124 residents from the five largest metro areas in Texas. Table 2 summarizes demographic descriptive statistics for the sample. Rural and small-town communities ranged in population size from Bandera (pop. 910 in 2021) to Amarillo (pop. 199,747 in 2021) with an average of 24,692 and a median of 7325. The complete list of rural communities is available in the data supplement for this manuscript. The 2021 El Paso metro area population is 963,000. The other 4 metro (Dallas/Fort Worth, Houston, Austin, and San Antonio) areas each have 2021 populations well above 1 million.

**Table 2:** Sample Demographics

Gender (n = 5487)	Male	33.0%
	Female	67.0%
Race/Ethnicity (n = 5487)	White, not Hispanic or Latino	56.8%
	Hispanic or Latino	24.2%
	Black or African American	8.7%
	American Indian or Alaskan Native	1.0%
	Asian	5.5%
	Native Hawaiian or Pacific Islander	0.3%
	Two or more races	3.6%
Education (n = 5487)	Less than 9th grade	0.4%
	9th to 12th grade, without diploma	1.6%
	High school diploma or GED	12.6%
	Some college	21.7%
	Associate degree	10.5%
	Bachelor’s degree	30.1%
	Graduate or professional degree	23.1%
Age (n = 5487)	18–24	10.3%
	25–34	17.3%
	35–44	22.8%
	45–54	19.5%
	55–64	16.5%
	65–74	10.8%
	75 or older	2.8%
Household income (n = 5439)	Less than $10,000	4.8%
	$10,000 to $14,999	3.4%
	$15,000 to $24,999	5.9%
	$25,000 to $34,999	8.4%
	$35,000 to $49,999	11.3%
	$50,000 to $74,999	17.9%
	$75,000 to $99,999	15.8%
	$100,000 to $149,999	18.1%
	$150,000 to $199,999	7.7%
	$200,000 or more	6.7%

## Data Analysis Strategy

Descriptive statistics as well as exploratory factor and reliability analyses were conducted using SPSS 27.0. Generalized linear mixed models were used to answer our research questions. Each model was fit with a logit link function and binomial distribution. Interest in each project concept was coded as yes (allocation of any points) or no (allocation of zero points). Due to the multiple sites involved in this study, interest in each project concept was modeled using random intercept mixed models. All predictive models were run in R with the glmmTMB package 1.0.2.1 (Brooks, et al. 2017).

Each model was tested in a series of steps: first, community characteristics were entered, followed by social position and COVID impact; additional steps added the independent variables for values, personality, and asset satisfaction and importance. The improvement of the models with the addition of variables after each step was assessed with the reduction in AIC. In summary, the specification for the models employed in the present study is shown in Table 3.

**Table 3:** Variables Included in Project Concept Interest Models

Dependent Variables
Interest (0/1) in each of: • Renovating some downtown buildings as mixed-use facilities with retail shops and apartments • Opening a community health center • Deploying gigabit high-speed fiber broadband internet downtown
﻿Stage 1: Community – Research Question 1
• Population (numeric, log(population)) • Population Density (numeric, people/square mile) • Distance to the nearest major metro area (numeric, km)
Stage 2: Social Position - Research Question 2 (and COVID Controls)
• Age (numeric, with the value taken at the lower end of each categorical range) • Gender (reference = male) • Minority Race/Ethnicity (reference = White) • Education (Reference = Less than college degree) • COVID impact on community’s health (numeric, 1–7 agreement rating) • COVID impact on community’s economy (numeric, 1–7 agreement rating)
Stage3: Predictors for Research Question 3
• Resultant Self-Transcendence, Self-Transcendence - Self Enhancement (numeric, difference between two 1–7 subscale mean agreement ratings) • Resultant Conservation, Conservation - Openness to Change (numeric, difference between two 1–7 subscale mean agreement rating) • Agreeableness (numeric, 1–7 subscale mean agreement rating) • Openness (numeric, 1–7 subscale mean agreement rating)
Stage 4: Predictors for Research Questions 4
• Satisfaction with broad-based community services (numeric, 1–7 mean rating) • Satisfaction with economic environment (numeric, 1–7 mean rating) • Satisfaction with family-oriented community services (numeric, 1–7 mean rating) • Satisfaction with communications services (numeric, 1–7 mean rating) • Importance of broad-based community services (numeric, 1–7 mean rating) • Importance of economic environment (numeric, 1–7 mean rating) • Importance of family-oriented community services (numeric, 1–7 mean rating) • Importance of communications services (numeric, 1–7 mean rating)

## Results

Table 4 summarizes the hierarchal model results for the three community development project concepts, including fit statistics for each model as information is added to the hierarchy. The table includes the Akaike information criteria (AIC) for each model, the change in the *Χ*^2^ statistic as information is added, and the significance of the change in model fit.

More specifically, Table 4 shows the change in model fit when the following stages of information are added to the models for each community development project concept: (1) a random intercept is included to control for variability across the sites in the study, (2) three location variables for each community, (3) social position and community perceptions of the impact of COVID on the community, (4) four variables for personal values and personality, and (5) eight asset satisfaction and importance variables. The inclusion of the random intercept improved fit for all models. The sequential addition of location variables significantly improved fit for the model for the community health center (p < 0.009). (The improvement in fit for gigabit fiber was significant at p = 0.055.) Fit was improved significantly after the addition of social position and community perceptions of the impact of COVID for all models; all significant at p < 0.001. The addition of personal values and personality variables significantly improved the fit of the model for downtown renovation (p < 0.001), but did not improve the fit of the models for opening a community health center or adding gigabit fiber downtown. The fit of all three models was improved by the addition of information about perceptions about satisfaction and importance of community assets, all significant at p ≤ 0.001.

**Table 4:** Hierarchical Modeling Summary

Downtown Renovation for Mixed Use Facilities					
	**df**	**AIC**	**Δ** ***Χ*** ^**2**^	**Δ** ***Χ*** ^**2**^ **df**	**Sig.**
Intercept	1	5228.6			
Random Intercept	2	5109.3	121.3	1	0.000
Location	5	5112.4	2.9	3	0.406
Social Position	11	5096.3	28.1	6	0.000
Values & Personality	15	5066.1	38.2	4	0.000
Asset Satisfaction and Importance	23	5053.2	29.0	8	0.004
**Community Health Center**					
	**df**	**AIC**	**Δ** ***Χ*** ^**2**^	**Δ** ***Χ*** ^**2**^ **df**	**Sig.**
Intercept	1	5695.0			
Random Intercept	2	5523.6	173.4	1	0.000
Location	5	5518.1	11.5	3	0.009
Social Position	11	5487.5	42.6	6	0.000
Values & Personality	15	5492.8	2.7	4	0.606
Asset Satisfaction and Importance	23	5425.7	83.1	8	0.000
**Gigabit Fiber Broadband Downtown**					
	**df**	**AIC**	**Δ** ***Χ*** ^**2**^	**Δ** ***Χ*** ^**2**^ **df**	**Sig.**
Intercept	1	6569.5			
Random Intercept	2	6430.4	141.1	1	0.000
Location	5	6428.8	7.6	3	0.055
Social Position	11	6381.2	59.7	6	0.000
Values & Personality	15	6384.7	4.5	4	0.348
Asset Satisfaction and Importance	23	6294.9	105.8	8	0.000

Tables 5a, 5b, and 5c show the detailed results for each community development project concept as perceived by communities, and include the adjusted odds ratio (AOR), standard error (SE), and the significance (Sig.) for all terms in each model. We focus our discussion on model effects (AOR) that are significant at 0.05 or less, and organize this discussion around our research questions. The presentation of these results will start with the results from the hierarchical modeling process across all three concepts followed by a presentation of the results for our research questions for each model taken in turn: question 1 (community characteristics), 2 (social position), 3 (personal values and personality types), and 4 (asset satisfaction and importance).

The discussion of these models focuses on the final, or full, model. Only one change in the structural results were observed as stages of information were added to the hierarchy. Specifically, for the *community health center model*, age dropped from significance in the final model, but earlier stages indicated that increased age predicted lower interest in this concept.

*Downtown renovation for mixed use facilities.* As shown in Table 5a, increasing age predicts a lower likelihood of interest in a downtown renovation for mixed use facilities (AOR = 0.993, p = 0.006). A more agreeable personality predicts a higher likelihood of interest in a downtown renovation for mixed use facilities (AOR = 1.110, p = 0.017). Greater resultant self-transcendence predicts a lower likelihood of interest in a downtown renovation for mixed use facilities (AOR = 0.902, p < 0.001). Higher satisfaction with available broadband and cellular/mobile predicts a higher likelihood of interest in a downtown renovation for mixed use facilities (AOR = 1.076, p = 0.005).

*Community health center*. As shown in Table 5b, minority status predicts a higher likelihood of interest in a community health center (AOR = 1.311, p = 0.002). Perceiving a greater health impact of COVID on one’s community predicts a higher likelihood of interest in a community health center (AOR = 1.088, p = 0.012). Additively, lower satisfaction with and greater perceived importance of available family-oriented community services predict a higher likelihood of interest in a community health center, respectively (AOR = 0.814, p < 0.001) and (AOR = 1.191, p < 0.001). Additively, higher satisfaction with and lower perceived importance of available broadband and cellular/mobile predict a higher likelihood of interest in a community health center (AOR = 1.104, p < 0.001) and (AOR = 0.993, p = 0.009). Greater perceived importance of broad-based community services predicts a higher likelihood of interest in a community health center (AOR = 1.159, p = 0.001). Greater perceived importance of the economic environment predicts a lower likelihood of interest in a community health center (AOR = 0.833, p < 0.001).

*Adding gigabit fiber to a downtown area*. As shown in Table 5c, increased distance from a major metro area predicts a lower likelihood of interest in adding gigabit fiber to a downtown area (AOR = 0.999, p = 0.034). Female community members are less likely than males to be interested in adding gigabit fiber to a downtown area (AOR = 0.787, p = 0.001). Community members with at least an undergraduate degree are more likely than community members with less education to be interested in adding gigabit fiber to a downtown area (AOR = 1.420, p < 0.001). Additively, lower satisfaction with and higher perceived importance of available broadband and cellular/mobile predict a higher likelihood of interest in adding gigabit fiber to a downtown area (AOR = 0.839, p < 0.001) and (AOR = 1.213, p < 0.001). Higher perceived importance of broad-based community services predict a lower likelihood of interest in adding gigabit fiber to a downtown area (AOR = 0.924, p = 0.048).

**Table 5:** a. Downtown Renovation for Mixed Use Facilities Concept Interest Model Results (Fixed Effects from GLMM Logistic with RI)

	Location			+ Identity			+ Values & Personality			+ Asset Satisf. & Importance		
	**AOR**	**SE**	**Sig.**	**AOR**	**SE**	**Sig.**	**AOR**	**SE**	**Sig.**	**AOR**	**SE**	**Sig.**
Intercept	3.383	0.20	0.000	6.451	0.26	0.000	4.920	0.31	0.000	3.719	0.44	0.003
Log Population (000)	0.942	0.06	0.346	0.927	0.06	0.228	0.920	0.06	0.181	0.925	0.06	0.215
Population Density	1.000	0.00	0.264	1.000	0.00	0.295	1.000	0.00	0.332	1.000	0.00	0.451
Distance to nearest Urban Center (km)	1.001	0.00	0.281	1.000	0.00	0.330	1.000	0.00	0.335	1.000	0.00	0.434
Age				0.989	0.00	0.000	0.993	0.00	0.005	0.993	0.00	0.006
Female				0.841	0.08	0.029	0.917	0.08	0.288	0.931	0.08	0.382
Minority				0.941	0.09	0.492	0.964	0.09	0.684	0.968	0.09	0.721
Undergrad degree or more				1.134	0.07	0.089	1.115	0.08	0.145	1.082	0.08	0.295
COVID health impact				1.047	0.04	0.197	1.046	0.04	0.208	1.050	0.04	0.171
COVID economic impact				0.973	0.04	0.460	0.976	0.04	0.523	0.992	0.04	0.836
Agreeableness							1.103	0.04	0.022	1.110	0.04	0.017
Openness							0.996	0.04	0.923	1.001	0.04	0.982
Self-Transcendence – Self-Enhancement							0.902	0.02	0.000	0.902	0.02	0.000
Conservation - Openness to Change							0.969	0.02	0.188	0.968	0.02	0.186
Sat. with broad-based community services										0.887	0.04	0.004
Sat. with economic environment										1.063	0.04	0.102
Sat. with family-oriented community services										1.084	0.04	0.066
Sat. with broadband and cellular/mobile										1.076	0.03	0.005
Imp. of broad-based community services										1.065	0.05	0.167
Imp. of economic environment										0.918	0.05	0.081
Imp. of family-oriented community services										1.030	0.05	0.532
Imp. of broadband and cellular/mobile										0.965	0.03	0.269

Caption: AOR = Adjusted Odds Ratio, SE = Standard Error, Sig. = Significance

**Table 5:** b. Community Health Center Concept Interest Model Results (Fixed Effects from GLMM Logistic with RI)

	Location			+ Identity			+ Values & Personality			+ Asset Satisf. & Importance		
	**AOR**	**SE**	**Sig.**	**AOR**	**SE**	**Sig.**	**AOR**	**SE**	**Sig.**	**AOR**	**SE**	**Sig.**
Intercept	1.877	0.17	0.000	1.968	0.24	0.004	1.847	0.29	0.033	1.927	0.41	0.110
Log Population (000)	1.118	0.06	0.055	1.085	0.06	0.160	1.085	0.06	0.158	1.068	0.06	0.262
Population Density	1.000	0.00	0.834	1.000	0.00	0.888	1.000	0.00	0.912	1.000	0.00	0.945
Distance to nearest Urban Center (km)	1.000	0.00	0.925	1.000	0.00	0.829	1.000	0.00	0.831	1.000	0.00	0.551
Age				0.995	0.00	0.020	0.994	0.00	0.013	0.996	0.00	0.101
Female				1.044	0.07	0.560	1.018	0.08	0.812	1.009	0.08	0.907
Minority				1.403	0.09	0.000	1.399	0.09	0.000	1.311	0.09	0.002
Undergrad degree or more				1.026	0.07	0.718	1.029	0.07	0.684	1.040	0.07	0.585
COVID health impact				1.105	0.03	0.002	1.107	0.03	0.002	1.088	0.03	0.012
COVID economic impact				0.986	0.04	0.683	0.988	0.04	0.723	0.992	0.04	0.818
Agreeableness							0.981	0.04	0.628	0.971	0.04	0.475
Openness							1.034	0.04	0.397	1.065	0.04	0.128
Self-Transcendence – Self-Enhancement							1.027	0.02	0.185	1.015	0.02	0.455
Conservation - Openness to Change							1.001	0.02	0.962	1.004	0.02	0.865
Sat. with broad-based community services										1.063	0.04	0.113
Sat. with economic environment										1.006	0.04	0.858
Sat. with family-oriented community services										0.814	0.04	0.000
Sat. with broadband and cellular/mobile										1.104	0.02	0.000
Imp. of broad-based community services										1.159	0.04	0.001
Imp. of economic environment										0.833	0.05	0.000
Imp. of family-oriented community services										1.191	0.05	0.000
Imp. of broadband and cellular/mobile										0.923	0.03	0.009

Caption: AOR = Adjusted Odds Ratio, SE = Standard Error, Sig. = Significance

**Table 5:** c. Gigabit Fiber Downtown Concept Interest Model Results (Fixed Effects from GLMM Logistic with RI)

	Location			+ Identity			+ Values & Personality			+ Asset Satisf. & Importance		
	**AOR**	**SE**	**Sig.**	**AOR**	**SE**	**Sig.**	**AOR**	**SE**	**Sig.**	**AOR**	**SE**	**Sig.**
Intercept	1.968	0.16	0.000	1.696	0.22	0.015	1.861	0.26	0.018	1.711	0.37	0.144
Log Population (000)	0.979	0.05	0.695	0.971	0.05	0.579	0.973	0.05	0.599	0.985	0.05	0.769
Population Density	1.000	0.00	0.431	1.000	0.00	0.370	1.000	0.00	0.376	1.000	0.00	0.147
Distance to nearest Urban Center (km)	0.999	0.00	0.008	0.999	0.00	0.006	0.999	0.00	0.006	0.999	0.00	0.034
Age				1.004	0.00	0.058	1.005	0.00	0.031	1.003	0.00	0.200
Female				0.805	0.07	0.001	0.807	0.07	0.002	0.787	0.07	0.001
Minority				0.964	0.08	0.634	0.969	0.08	0.677	0.955	0.08	0.551
Undergrad degree or more				1.422	0.06	0.000	1.407	0.06	0.000	1.420	0.07	0.000
COVID health impact				1.072	0.03	0.022	1.070	0.03	0.025	1.062	0.03	0.050
COVID economic impact				0.930	0.03	0.024	0.928	0.03	0.021	0.918	0.03	0.009
Agreeableness							0.958	0.04	0.239	0.947	0.04	0.153
Openness							1.008	0.04	0.822	1.012	0.04	0.750
Self-Transcendence – Self-Enhancement							0.993	0.02	0.710	0.996	0.02	0.844
Conservation - Openness to Change							0.964	0.02	0.070	0.964	0.02	0.082
Sat. with broad-based community services										1.036	0.04	0.319
Sat. with economic environment										0.991	0.03	0.789
Sat. with family-oriented community services										0.989	0.04	0.762
Sat. with broadband and cellular/mobile										0.839	0.02	0.000
Imp. of broad-based community services										0.924	0.04	0.048
Imp. of economic environment										1.011	0.04	0.789
Imp. of family-oriented community services										1.003	0.04	0.941
Imp. of broadband and cellular/mobile										1.213	0.03	0.000

Caption: AOR = Adjusted Odds Ratio, SE = Standard Error, Sig. = Significance

## Discussion

The present study illustrates that interest in various community development project concepts is differentially and variously correlated with many factors, including the identities of community members, their personalities, and personal values, as well as their levels of satisfaction with and the importance of the assets that are available in their community. However, the patterns seen in the connections among these factors, and interest in community development projects, differ little along degrees of urbanity and rurality as measured in the present study, though they do hint at how relationships among communities may effect these processes.

Agreeableness is positively associated with interest in a downtown renovation for mixed-use facilities, while resultant self-transcendence is negatively associated with interest in the same community development concept. Neither is associated with interest in a community health center or adding Gigabit fiber. Satisfaction with broadband and cellular/mobile services is connected to interest in a downtown renovation for mixed-use facilities.

Interest in a community health center is connected to minority status. However, interest is also connected to satisfaction with and perception of the importance of several community assets. In particular, lower levels of satisfaction and higher perceptions of importance for family-oriented community services are connected to interest in a community health center. Interestingly, higher levels of satisfaction and lower perceptions of importance for broadband and cellular/mobile are connected to interest in a community health center.

These results highlight the necessity of including local perspectives in sustainably developing a community, in agreement with the SLED model. Additionally, these results illustrate that decision-making about community development is connected to both emotional perspectives held by community members and their rational assessment of the performance of assets in their community. Further, the role of these emotional and rational decision drivers varies by the context of the decision being considered.

Findings presented here reinforce the complex understanding of the interconnectedness among community, personality, and satisfaction/importance variables in community economic development presented in the SLED and ABCD frameworks. Both approaches deal with individuals, relationships, and organizations to optimize community development decision-making. This paper has shown that personality traits, too, affect the relationships people form, their motivations for engaging with others, and the types of activities in which they engage (community development being one such activity).

While community-level characteristics had a limited impact on development project preferences – distance from an urban center decreasing support for the broadband intervention – their qualified significance and inclusion in this study went beyond the frameworks of ABCD and SLED speaking to the relationship communities have to each other. Part of ABCD is the creation of communities through interaction. Knowing that a community is close to an urban center where there are generally centralized assets, resources and services could tend to make one feel just as connected to that center as their home community, and thus not see their community as lacking. Population size and density also increases individuals’ ability to interact with others, therefore making different types of development activity possible, increasing the assets in play. If communities are far from urban centers, or have low populations that are spread apart from one another, then interaction among community members is harder and there will be more gaps in agreement on what improvements in which to invest. The models and data presented here only partially confirm this intuition, and further research on community distance from major urban metropolitan areas, community identity, and gaps in satisfaction and importance should be further developed.

## Limitations

The present study is not without limitations. Data collection during the COVID-19 pandemic limited our ability to sample small town and rural communities, despite the community outreach methods we utilized, described earlier. Although we utilized social media; telephone calls to local community members and leaders; and distributing electronic fliers to local groups to post on their websites, future research, conditions permitting, should include deeper in-person outreach into selected communities to ensure better representation of marginalized community members who are more likely to have faced systematic barriers and inequalities. Such deeper human-centered engagement would allow researchers to establish greater degrees of trust in the community and to connect with community members in the places where they physically gather, including places of worship, town squares, public parks, etc. where both social networking to establish trust and intercept interviewing to increase randomness of participation would likely extend the electronic outreach used in this study during the pandemic.

Additionally, as discussed above, the present study operationalized degree of urbanity/rurality with only 3 measures, community population size, population density, and distance from one of the 5 major metropolitan areas in the state. Future research should more systematically select variables that capture variation across the urban-rural continuum (from the spatial, cultural, ecological, demographic levels) as well as test which competing sets of variables provide the most explanatory power for the processes being studied.

Lastly, the present study addressed the consideration aspect of thinking about community development project concepts. Although our survey included an allocation exercise, analysis of and predictions about the actual allocation of points was left to a subsequent manuscript. Mimicking the process of making budget or resource allocations, that point allocation analysis will examine the trade-offs made by community members as they decide the quantity of points to give to each concept that they deem worthy of consideration. Such an analysis will need to address the compositional nature of the allocation (Wang, et al. 2013; Tsagris 2015); Feng et al. 2017; Fry, Fry, & McLaren 2000). Further, hurdle models are ideally suited for assessing this kind of sequential decision-making process, first modeling the consideration step and then, conditioned on consideration, modeling the allocation (Kammer-Kerwick, et al. 2019; Ntuli, et al. 2021; and Zuur, et al. 2011). Of particular interest in application of this approach is whether or not a different set of predictors are better suited to improving understanding of the allocation step than those that predict the consideration step (Morris, et al. 2021).

## Conclusions

This analysis clarifies the complex role of community characteristics, the personal values and personality types of members of those communities, and the assessment by community members of their satisfaction with and the importance that they perceive for various assets available in the community. All of these factors have the potential to influence interest in different community development concepts, but they also do so differently across concepts. These nuanced results reinforce the benefits of incorporating local community perspectives in community development planning and approaching the development process as purposefully addressing a system of subsystems (Nel 2018). Indeed, different community development concepts fill the needs of different community segments because they enhance or expand different subsystems. The findings presented here also locate opportunities to engage in further practical interventions within the community development process. Our findings specifically highlight areas where human-centered, participatory processes can be brought in to guide further interactions between experts and community members to (1) identify groups in the community to bring into the process who may be most affected by the choice of one intervention over another, as well as (2) target areas where further community discussion and deliberation is necessary around which community dialogues (round tables, town halls, workshops, etc.) could be facilitated.

The first type of practical intervention seeks to identify who in the community is most interested in a given type of community development project in order to get both an idea of who the project is serving as well as who should be targeted for outreach and brought further into the development process. The findings related to the Community Health Center project intervention showed that minority-status was an outsized predictor for gauging levels of support for this intervention. In this instance, minorities supported establishing a Community Health Center more than white community members, which makes sense given the large disparities that exist in healthcare provision along racial lines. Minorities have historically been left out of community development decision-making and have been the most under-served by development interventions as a result, making the benefits of this development project to minority community members especially impactful. These concerns over inclusion and exclusion find themselves reappearing along different lines across community development processes, which should push researchers and practitioners to step back and ask about who is being represented in the decision-making process, whether they are representative of the community as a whole, and whether the right voices and perspectives have been engaged. The success of a community choosing such a project relies on targeting and engaging various groups of community members in the decision-making process through outreach.

The second type of intervention seeks to identify and target specific domains of intervention where community preferences and priorities are still inchoate and could benefit from further discussion and deliberation. The findings related to the broadband project intervention above reveal that while experts have repeatedly shown that rural broadband is extremely important for both community wellbeing and economic growth, support for broadband interventions declined among our respondents the farther they lived from an urban center. This may be a situation where local perceptions are a barrier to implementing community development initiatives that are known to produce positive and outsized benefits in just such a community. In such a situation, a round table could be planned where experts and members of other similar communities that implemented broadband projects could share how their communities benefitted from broadband and share the struggles they faced in implementing their projects. The goal of facilitating such a community dialogue would be to make community members aware of the unknowns of a given project while also encouraging discussion and collaboration across communities to this end, forming relationships that could support and guide the community development process moving forward.

The development of both types of interventions call for a participatory action research process (Baum, MacDougall, and Smith 2006) that brings community members into discussions with policy makers, development professionals, and researchers at the very beginning of planning and deliberation about options that allow the design to emerge with input from all (Genat 2009).

This analysis of community development priorities across geographies in a large U.S. state reveals more commonalities than differences between urban and rural residents. Surprisingly, preferences among economic and quality of life development choices did not break cleanly into an urban-rural binary but were dependent on a far more complex set of identities. Economic development professionals would be well advised to acknowledge this complexity in their communities when weighing the choices available to them, for the well-being of their whole community.

## Electronic Supplementary Material

Below is the link to the electronic supplementary material.

Supplementary Material 1

## Footnotes

1 Dynata.com.
