# Supplementary material for: Asset-Based, Sustainable Local Economic Development: Using Community Participation to Improve Quality of Life Across Rural, Small-Town, and Urban Communities
Source: Appl Res Qual Life. 2022 Jun 22;17(5):3023–47. doi: 10.1007/s11482-022-10051-1 (PMC9213640; doi:10.1007/s11482-022-10051-1)
Supplement: Supplementary file 1 — Supplementary Material 1 [file 11482_2022_10051_MOESM1_ESM.docx]

Response to Reviewers for ARIQ-D-21-00935

Asset-Based, Sustainable Local Economic Development: Using Community Participation to Improve Quality of Life Across Rural, Small-Town, and Urban Communities

March 4, 2022

Daniel Shek, PhD

Editor in Chief

Applied Research in Quality of Life

Dear Dr. Shek,

I want to thank you and the reviewers for your valuable feedback on our manuscript, ARIQ-D-21-00935, and I am pleased to submit this revision for your review. Our research team has addressed the specific feedback received and have looked for ways to improve our manuscript beyond those specific points in ways that we believe are implied by the suggestions.

1. Explain the meaning of "human-centered approach"
2. The finding that a wide range of factors are related to perceived needs is not novel. Elaborate more on the theoretical and practical significance of the findings.
3. Elaborate more on the "participatory process".
4. The paper lacks a critical flavor. What are the limitations of the study?

We address each of these in turn.

**#1 - “human-centered approach” and #3 “participatory process”**

We believe that points 1 and 3 are connected and have addressed them in multiple locations in the manuscript with clarifying narrative and additional references to literature. Specifically:

Page 2, paragraph 2, we have added the sentence:

By using a participatory process to consider what members of different communities would choose for their communities to invest in using a trade-off exercise, we put the human and community member view of their needs first in determining which projects should be prioritized in any given community, looking at how each community member sees the connected needs of the community and how they impact each other.

We have also revised the second paragraph on page 3 to read:

We focus on a human-centered, participatory approach that builds from SLED and ABCD by attempting to start from the needs of the community as a way of assessing projects that would meet the human needs of the members of that community. Good jobs, human flourishing, and community creation are as important—if not more important—than economic growth. This framing of a human-centered approach to sustainable community development draws from the literature on human-centered design, wherein product solutions are created with the involvement of the human perspective at all steps in the planning and development process. However, this approach is less commonly applied to psychosocial interventions and implementation strategies (Lyon, Brewer, & Areán 2020). Here, the quality of life of the community is a way to plan for sustainable and locally appropriate economic development that requires individual-, community-, and economy-level information.

Last, in the conclusion (page 18, paragraph 3), we connect our approach to studying community development to participatory action research

The development of both types of interventions call for a participatory action research process (Baum, MacDougall, and Smith 2006) that brings community members into discussions with policy makers, development professionals, and researchers at the very beginning of planning and deliberation about options that allow the design to emerge with input from all (Genat 2009).

**#2 - wide range of factors are related to perceived needs is not novel**

We have extended our discussion of this finding on page 16 by tapping into relevant literature and adding the following to paragraph 5 on that page.

Farinosi, Fortunati, O’Sullivan, & Pagani (2019) describe public participation in democracy on three levels, the information level, advisory level, and deliberative level. By distributing a survey through outreach to residents in rural and small-town communities, our research demonstrates the possibilities of listening to citizens as “active subjects of knowledge and expertise.” As such, we contribute to a rich literature on how to navigate the complexity of sustainable community development through participatory methods (e.g., Gomes, Hermans, and Thissen, 2017; Kerselaers, Rogge, Vanempten, Lauwers, & Van Huylenbroeck, 2013; and Valencia-Sandoval, Flanders, & Kozak, 2010) with an examination of the role of a specific set of human-centered variables. By showing that there is variation in perceived needs and tying this, in part, to urban-rural community and socio-demographic differences, we hope that we can motivate the field to identify commonalities between communities and community members that can be used to make more targeted and specific types of economic development plans and processes that can be applied across different contexts.

**#4 - What are the limitations of the study?**

Thank you for this feedback, a clear omission on our part. We have added a limitations section that begins at the bottom of page 16. We recognize that, despite our best efforts during the COVID pandemic, the degree of participation that we achieved in sampling from small town and rural communities is a limitation in the current study. We acknowledge this limitation and discuss more inclusive sampling methods that we planned to use here and will return to using in future research when in-person fieldwork is feasible. We also discuss some data and analysis limitations in the current study that are a function of the constraints on length and complexity of a single paper. We discuss how these limitation can be addressed as part of our future research plan.

**Reviewer Comments**

We have corrected both of the errors noted by the reviewer.

P.2, 2nd line under the heading, 'The Role of Community and Q..o..L in Economic Development'. (Delete comma and extra space in line starting with:) 'and decision-making criteria, . Sustainable…..'

P.4, last two lines under heading of 'Method': 'by the degree of satisfaction …. as well as how important those resources (insert 'are') to the community.'

We have added language clarifying the meaning of “K-12 education” in the US for readers not familiar with that vernacular.

P. 5. This reader was not familiar with 'K-12 education'. Google informed her that K-12 refers to some free education in the US. Possibly, a footnote might be helpful for international readers as free elementary education is also a concern for citizens in other countries, particularly in less developed ones.

Thank you, again, for the time and effort spend reviewing our manuscript. We appreciate the feedback and your contribution to our paper.
